# Testis exposure to unopposed/elevated activin A *in utero* affects somatic and germ cells and alters steroid levels mimicking phthalate exposure

**DOI:** 10.3389/fendo.2023.1234712

**Published:** 2023-09-01

**Authors:** Penny A. F. Whiley, Michael C. M. Luu, Liza O’Donnell, David J. Handelsman, Kate L. Loveland

**Affiliations:** ^1^ Centre for Reproductive Health, Hudson Institute of Medical Research, Clayton, VIC, Australia; ^2^ Department of Molecular and Translational Sciences, School of Clinical Sciences, Monash University, Clayton, VIC, Australia; ^3^ ANZAC Research Institute, University of Sydney, Concord, NSW, Australia

**Keywords:** spermatogenesis, gonocyte, testicular dysgenesis, MEHP, phthalate, multinucleated germ cell, inhibin

## Abstract

Correct fetal testis development underpins adult male fertility, and TGFβ superfamily ligands control key aspects of this process. Transcripts encoding one such ligand, activin A, are upregulated in testes after sex determination and remain high until after birth. Testis development requires activin signalling; mice lacking activin A (*Inhba* KO) display altered somatic and germ cell proliferation, disrupted cord elongation and altered steroid synthesis. In human pregnancies with pre-eclampsia, the foetus is inappropriately exposed to elevated activin A. To learn how this affects testis development, we examined mice lacking the potent activin inhibitor, inhibin, (*Inha* KO) at E13.5, E15.5 and PND0. At E13.5, testes appeared similar in WT and KO littermates, however E15.5 *Inha* KO testes displayed two germline phenotypes: (1) multinucleated germ cells within cords, and (2) germ cells outside of cords, both of which are documented following *in utero* exposure to endocrine disrupting phthalates in rodents. Quantitation of Sertoli and germ cells in *Inha* KO (modelling elevated activin A) and *Inhba* KO (low activin A) testes using immunofluorescence demonstrated activin A bioactivity determines the Sertoli/germ cell ratio. The 50% reduction in gonocytes in *Inha* KO testes at birth indicates unopposed activin A has a profound impact on embryonic germ cells. Whole testis RNAseq on *Inha* KO mice revealed most transcripts affected at E13.5 were present in Leydig cells and associated with steroid biosynthesis/metabolism. In agreement, androstenedione (A4), testosterone (T), and the A4:T ratio were reduced in *Inha* KO testes at E17.5, confirming unopposed activin A disrupts testicular steroid production. E15.5 testes cultured with either activin A and/or mono-2-ethylhexyl phthalate (MEHP) generated common histological and transcriptional outcomes affecting germline and Leydig cells, recapitulating the phenotype observed in *Inha* KO testes. Cultures with activin A and MEHP together provided evidence of common targets. Lastly, this study extends previous work focussed on the *Inhba* KO model to produce a signature of activin A bioactivity in the fetal testis. These outcomes show the potential for elevated activin A signalling to replicate some aspects of fetal phthalate exposure prior to the masculinization programming window, influencing fetal testis growth and increasing the risk of testicular dysgenesis.

## Introduction

Proper testis development in fetal life is central to male reproductive health. *In utero* disruptions due to physiological insults or exposure to environmental toxins can lead to inappropriate cell signalling that impairs somatic and germ cell function. This increases the risk for a range of reproductive disorders including hypospadias, cryptorchidism, testicular cancer and infertility, collectively termed Testicular Dysgenesis Syndrome, TDS (1). Because the cell signalling inputs required for normal development are vulnerable to physiological and environmental insults, it is important to consider what pathways are activated in pregnancy that may contribute to increasing rates of TDS phenotypes.

In this study, we focus on activin A, a highly conserved growth factor in the TGF-β superfamily (100% identical between human and mouse). It is central to fetal testis development, and its levels are increased during human pregnancy under certain pathological conditions. Elevated activin A in maternal serum during pregnancy is an indicator of both pre-eclampsia and fetal growth restriction (2, 3), which each affect an estimated 3-8% of all pregnancies. In pregnancies complicated by intraamniotic infection, activin A levels in the amniotic fluid are increased, and correlate directly with infection severity (4). Activin A is significantly elevated in both maternal and fetal cord blood of women taking selective serotonin re-uptake inhibitors (SSRIs) (5). Thus, activin A can be elevated in several pregnancy complications, and the present study begins to address the knowledge gap regarding whether these conditions can ultimately influence testis development or male fertility.

Studies conducted with mice have demonstrated crucial roles for activin A in fetal testis growth. Testes first develop after sex determination at E11.5 in mice, accompanied by elevation of transcripts encoding activin A (*Inhba*), which peak at birth (6). Conversely, in fetal ovaries, *Inhba* levels remain low and its potent antagonist, follistatin, is produced (7). The rise in *Inhba* transcripts coincides with development of the testis cords and production of factors, including hormones, which enable masculinization of the reproductive tract. During cord formation, emerging Sertoli cells enclose the germ cells (also termed gonocytes or pro-spermatogonia) to create the niche that enables spermatogenesis. This process is accompanied by recruitment, differentiation, and organisation of several somatic cell types (8–11), with the result that the surrounding interstitium becomes populated with fetal Leydig cells, immune cells, fibroblasts, lymphatics and vasculature (12–14). Between E13.5 – E15.5, the mitotically active germ cells become quiescent, and a subset of apoptosis-poised germ cells undergo a wave of programmed cell death, considered to select for germline reproductive fitness (15, 16). Ultimately, the Sertoli and peritubular myoid cells establish the basement membrane which forms a permanent boundary between the cords/seminiferous tubules and interstitium.

Fetal and adult Leydig cells emerge from a common early steroidogenic precursor following sex determination, as recently delineated by single cell RNAseq (17) and discussed in (18). Production of activin A by fetal Leydig cells predominantly affects Sertoli cells (12, 19) which in turn drives multiple aspects of fetal testis development. Mice lacking activin A (*Inhba* global knockout) exhibit reduced Sertoli cell numbers and proliferation and increased germ cell numbers at birth (6, 12), while Leydig cell-specific deletion of *Inhba* (*Amhr2^cre^* ^/+^; *Inhba* ^fl/-^) results in reduced cord coiling between E15.5 and birth (12), showing activin A promotes fetal Sertoli cell proliferation. The potent activin antagonist, inhibin, is formed by dimerization of an inhibin α subunit (encoded by *Inha*) with a β subunit (inhibin A is an α:βA dimer, inhibin B is an α:βB dimer). In postnatal and adult testes, Sertoli cell production of inhibin B regulates pituitary FSH production by antagonising activin's stimulatory effect on FSH production by gonadotropes. Pituitary-derived FSH can also influence Sertoli cell proliferation from late fetal development but is considered to have the greatest influence on Sertoli cell numbers around the time of puberty; FSH signalling in fetal life is not regarded as vital for Sertoli and Leydig cell development (20–24).

We previously showed that the absence of activin A alters the testis steroid milieu specifically through its actions in Sertoli cells (19). Fetal Sertoli cells are the sole cellular site of transcripts (*Hsd17b1*, *Hsd17b3*) encoding the enzymes that convert androstenedione (A4) into testosterone (T). In *Inhba* KO mouse testes, their levels are markedly reduced, as is the level of T (19, 25, 26). These findings demonstrate the importance of activin A bioactivity to normal testosterone production during a critical period of embryo growth.

Perturbed androgen synthesis in the fetal testis can also be caused by exposure to endocrine disruptor chemicals (EDCs). Rodents exposed *in utero* to mono-ethylhexyl phthalate (MEHP), a phthalate ester commonly used as a plasticiser, during the masculinization programming window (MPW; E15.5-E18.5 in rats and weeks 8-11 in humans) interrupts both testicular development and androgen synthesis (27, 28). EDC exposure and the resulting disruption to steroidogenesis cause a range of phenotypes in the developing testis, including altered seminiferous cord formation and the emergence of multinucleated gonocytes (MNG). EDCs have indirectly, but not experimentally, been linked to an increased risk of male infertility and testicular cancer in humans (29, 30).

Although we previously showed that activin A deficiency disturbs fetal male gonad development and steroidogenesis, whether the reciprocal condition of elevated activin A influences fetal testis development is unknown. Given the range of pregnancy conditions linked with elevated activin A, it is important to identify the consequences of exposure to inappropriately high activin A for testis development. This study employs an established mouse model lacking the selective activin A inhibitor, inhibin (encoded by *Inha*), that features unopposed activin A, with chronically elevated activin A measured in adults (31, 32). We examined *Inha* WT and KO mouse testes at E13.5, E15.5 and PND 0, and compared them to *Inhba* WT and KO testes. The results reveal dose-dependent responses to activin A on gene expression and steroid production during the early stages of gonad masculinisation. The observation of phenotypes in *Inha* KO testes that are common to phthalate/endocrine disrupting chemical exposure highlight the potential for a functional relationship between activin A and MEHP which could determine male reproductive tract development and adult fertility.

## Materials and methods

### Animals

Two established mouse lines formed the basis of this work: *Inha* KO, lacking the *Inha* mature subunit coding sequence (31) and *Inhba* KO (BA 192), which lacks the *Inhba* mature subunit coding sequence (33). Both were maintained on a C57/Bl6 background. In addition, we utilised the *Inha* x OG2 (bearing Oct4-GFP) mouse strain described previously (19). All three lines were maintained by heterozygote breeding, and genotypes were determined by commercial vendor using real-time PCR (Transnetyx, TN, USA). Testes were collected from E13.5, E15.5 and PND 0 testes of *Inha* WT (wildtype) and KO littermates, E12.5, E13.5, E14.5 and E15.5 *Inhba* WT and KO littermates, and E15.5 WT and E17.5 WT and KO pups from the *Inha* x OG2 strain. For timed matings, the presence of a vaginal plug was used to determine embryonic day (E) 0.5 of pregnancy. Embryonic age was determined at the time of collection from fore- and hind-limb development and male gonads identified by the presence of testis cords. Natural birth, occurring on E19.5 – E20.5, was recorded as postnatal day 0 (PND 0). Testes were dissected free from mesonephros or epididymis at the time of collection. Mice were housed at the Monash Medical Centre Animal Facility in accordance with the Australian Code of Practice for the Care and Use of Animals for Scientific Purposes (1997), with a 12hr light/12hr dark cycle with food and water available ad libitum. This study was approved by the Monash University Animal Ethics Committee.

### Tissue collection

Testes were collected from E13.5 and E15.5 embryos and PND 0 pups. One testis was fixed in Bouin’s solution (Sigma-Aldrich) for 20 - 60 mins depending on age (E13.5: 20 mins, E15.5: 30 mins, PND 0: 60 mins), washed twice in 70% ethanol and stored at 4°C in 70% ethanol. Tissues were processed and embedded in paraffin wax using standard protocols by the Monash University Histology Platform (MHTP node), with 4 µm serial sections cut onto Superfrost™ Plus (Fisher Scientific) slides. The second testis was either fixed in 4% paraformaldehyde in PBS (PFA) (Alfa Aesar, Cat # J61899) for 40-90 mins depending on age (E13.5: 40 mins, E15.5: 60mins, PND 0: 90 mins) or snap-frozen and stored at -80°C for RNA. Incubations and washes were at room temperature (RT) unless otherwise indicated.

### Immunofluorescence

Sections on slides were dewaxed in histosol, then rehydrated in a series of decreasing ethanol concentrations (100% to 70%). All washes and incubations were at RT unless otherwise specified. Antigen retrieval was performed by microwaving slides in 50 mM glycine buffer (pH 3.5) for 10 minutes, then allowing them to cool for 20 mins. Tissue sections were blocked using 5% bovine serum albumin (BSA, Sigma-Aldrich, Cat # A7906) diluted in phosphate buffered saline (PBS, made from a 10X PBS stock, Gibco, Cat #70011-044) to prevent non-specific staining. All primary antibodies (DDX4, AF2030, R&D Systems, 1:400, RRID : AB_2277369; SOX9, Santa Cruz, sc-20095, 1:500, RRID : AB_661282; SMA, Sigma-Aldrich, A2547, 1:1000, RRID : AB_476701) were diluted in 1% BSA/PBS and applied overnight at 4°C. Negative control slides lacked primary antibodies. The next day, slides were washed 3 x 5 minutes in PBS, and sections were incubated with appropriate secondary antibodies (Alexa Fluor 488 donkey anti-mouse, Cat # A-21202, RRID : AB_141607; Alexa Fluor 488 donkey anti-goat, Cat # A-11055, RRID : AB_2534102; Alexa Fluor 546 goat anti-rabbit Cat # A-11010, RRID : AB_2534077, Alexa Fluor 546 rabbit anti-goat, Cat# A-21085, RRID : AB_1500595, all Invitrogen, 1:500) for 1 hour, then washed 3 x 5 minutes in PBS. Sections were mounted under coverslips with ProLong™ Gold Antifade Mountant with DAPI (Invitrogen, Cat # P36935) and stored at 4°C prior to image capture.

### Organ cultures

Whole E15.5 testis pairs from WT *Inha* x OG2 embryos were cut in half using a feather knife and placed on 30 mm diameter Millicell^®^ filters (Merck, Cat # PICM03050) in a 6 well plate, containing 0.6 mL of Dulbecco’s Modified Eagle Medium/F12 + Glutamax (Gibco, Cat # 105650-018) with 1% fetal calf serum (Bovogen) and 1% Pen/Strep (Gibco, Cat # 15670-063). Half testis fragments were cultured at 37°C in 5% CO_2_ in 25 ng/ml activin A (R&D Systems, Cat # AF338, RRID : AB_355307), 200 µm mono-2-ethylhexyl phthalate (MEHP; Santa Cruz, Cat # SC396467), 25 ng/ml activin A plus 200 µm MEHP, or appropriate vehicle control (4 μM HCl for activin A, DMSO for MEHP, or HCl plus DMSO, respectively). Testes from a single animal were used for both treatment and control groups. Media (50%) was refreshed daily. After 72 hours, fragments were fixed in Bouin’s for 1 hour and processed for histological analysis, or snap frozen on dry ice and stored at -80°C for transcriptional analyses.

### Imaging and counting strategy

Haematoxylin and immunohistochemistry images were collected using a light microscope (Olympus BX50 microscope with a DP70 camera). Immunofluorescence samples were scanned with a 40x objective, using the Olympus VS120^®^ Virtual Slide Microscope System at the Monash Histology Platform (MHTP node). Germ cells, Sertoli cells, cord area, and proliferating cells were identified in *Inha* and *Inhba* WT and KO mouse testes and in cultured testis fragment sections by immunofluorescence detection of DDX4 (gonocyte cytoplasm), SOX9 (Sertoli cell nuclei), and alpha-smooth muscle actin (SMA; peritubular cell cytoplasm, marking cord perimeter). Non-specific fluorescence signal was removed by setting the baseline exposure for section analysis equal to that of the negative control samples, which displayed no signal. Images were calibrated prior to analysis using ImageJ (RRID : SCR_003070) (34). From each testis cross section, the number of cords per 100 mm^2^, the average cord area in each section (*Inha* – E13.5: 15-30 cords, E15.5: 25-50 cords, PND 0: 50-100 cords; *Inhba* – E13.5: 15-30 cords, E15.5: 15-50 cords, PND 0: 15-100 cords), the total section area (mm^2^), and area of the section occupied by cords (mm^2^) were measured. Sertoli cell and gonocyte number were recorded for each section; the cross-section area, and area occupied by cords were used to normalise Sertoli and germ cell numbers. DDX4+ cells containing a visible nucleus both inside and outside cords were counted as gonocytes. Bi- and multinucleated gonocytes, identified by a DDX4+ cytoplasm surrounding closely opposed nuclei, were marked as abnormal and each was counted as a single cell.

### Fluidigm transcript analysis

*Inha* and *Inhba* fetal gonad age series and cultured E15.5 testes samples were measured in separate Fluidigm assays using the Fluidigm Biomark™ HD system (96×96 and 48x48 Dynamic Array IFC, respectively; Fluidigm Corporation) by the Monash Health Translation Precinct Medical Genomics Facility. For the *Inha* age series, whole, paired gonads from *Inha* WT and KO individual animals at E13.5, E15.5 and PND 0 (n=3/age/genotype) were collected from 2-3 litters. For the *Inhba* age series, paired gonads from *Inhba* WT and KO individual animals at E12.5, E13.5, E14.5 and E15.5 (n=3/age/genotype) were collected from 2-3 litters as previously described (19). Transcripts from E15.5 testis halves were measured following 72 hr culture with MEHP (200 µm) or vehicle control (n = 6-7/treatment). RNA extraction and on-column DNase-treatment was performed using the NucleoSpin RNA XS kit (Machery-Nagel) according to the manufacturer’s protocol. RNA quantity was determined using the Nanodrop (Thermo Scientific) and RNA quality was confirmed on a subset of samples using the Agilent 2100 Bioanalyzer (RNA integrity number (RIN) > 8.5). Reverse transcription of 300 ng (whole testes, *Inha* and *Inhba* age series) or 70 ng (cultured testis fragments) total RNA was performed using SuperScript™ III Reverse Transcriptase (Invitrogen) with random hexamer oligonucleotides (Promega), according to manufacturer’s guidelines. Fluidigm assays were performed using pre-designed and validated Taqman gene probes (Life Technologies). Raw data were analysed with Fluidigm Real-Time PCR Analysis Software (RRID : SCR_015686, Version 4.1.2). Negative control samples were below the detection limit. Both reference transcripts, *Canx* and *Mapk1* (31), were present at stable levels in all ages and genotypes, and an R value > 0.91 was obtained across all samples indicating they are positively correlated. On this basis, the average of both reference transcript values was used to normalize gene expression data using the ΔΔCT method (35).

### Steroid analysis

Snap frozen E17.5 paired testes from individual *Inha* x *OG2* WT and KO mice were homogenized in 130 µl ice cold homogenization buffer (0.5% BSA (w/v), 5 mM EDTA in PBS, pH 7.4) in a 5 ml glass tube for 20 seconds using an IKA T10 basic disperser on the highest setting; the probe was rinsed between samples in homogenisation buffer. Samples were centrifuged (3000 rpm, 10 min, 4°C) and supernatants transferred to new 1.5 ml plastic tubes for storage at -80°C until analysis. Steroid hormones were measured for all samples in a single batch using liquid chromatography–tandem mass spectrometry (LC–MS/MS) assay as previously described (19). The limit of detection (LOD) was set at the limit of quantification (LOQ) and represents the lower limit of assay sensitivity. Not detectable values were set at LOQ per analyte/2. The profile of steroids included androstenedione (A4), testosterone (T), 11-keto A4 (11-KA), 11-keto T (11-KT), 3α androstanediol (3α-diol), 3β androstanediol (3β-diol), dihydrotestosterone (DHT) and 11-keto DHT (11-KDHT), mirroring our previous analysis of the *Inhba* mouse testis at E17.5 (19).

### RNA-sequencing and bioinformatic analysis

Bulk RNA sequencing was performed on DNase-treated RNA from E13.5, E15.5 and PND 0 *Inha* WT and KO whole mouse testes (n = 2-3/age/genotype). Samples were sequenced by BGI-Hong Kong Co Ltd) on the DNBSEQ-G400 with PE100. Raw reads were filtered (using SOAPnuke software, BGI) to remove adaptor sequences, contamination and low-quality reads and stored in a FASTQ format. Bioinformatics analysis was performed at the Monash Bioinformatics Platform. Raw FASTQ files were analysed using the RNAsik (v1.5.4) pipeline (36) using STAR aligner (37) with GRCm38 (Mus musculus) reference genome. Reads were quantified using featureCounts (38) producing the raw genes count matrix and various quality control metrics. RNA-seq data are available via accession number: GSE236618. Raw counts were then analysed with Degust (39), a web tool which performs differential expression analysis using limma voom normalisation (40), producing counts per million (CPM) library size normalisation and trimmed mean of M values (TMM) normalisation (41) for RNA composition normalisation. Differentially expressed genes in *Inha* WT and KO testis at each age were defined as those with a false-discovery rate (FDR) of < 0.05, showing a > 3-fold (absolute log fold-change >1.5) change in expression. A heat map was generated using ClustVis (RRID : SCR_017133) (42). The list of DEGs was submitted to DAVID (RRID : SCR_001881) (43) to identify significantly associated functions. Venn diagrams were created using jvenn (RRID : SCR_016343) (44). Ingenuity Pathway Analysis software (IPA, RRID : SCR_008653) (Qiagen, Hilden, Germany) was used to analyse E13.5, E15.5 and P0 *Inha* WT and KO RNAseq data. Upstream regulators and gene networks were generated using IPA using stringency criteria p-value ≤0.05 and a Z-score of (inhibition) -3 ≤ Z ≥ 3 (activated). The Z-score is a prediction scoring system of activation or inhibition based upon statistically significant patterns in the dataset and prior biological knowledge manually curated in the Ingenuity Knowledge Base (45).

### Statistics

Statistical analysis for counting and Real time-PCR data was performed using GraphPad Prism software (RRID : SCR_002798). Distribution of data was determined using the Shapiro-Wilk normality test. Statistical differences between WT and KO data were determined using a two-tailed unpaired t-test. Data are expressed as mean ± SD. Statistical significance was set at * p<0.05, ** p<0.01, *** p<0.001, **** p<0.0001.

## Results

### Multinucleated gonocytes, and gonocytes outside the testis cords are features of Inha KO mouse fetal testes

**Figure 1:**
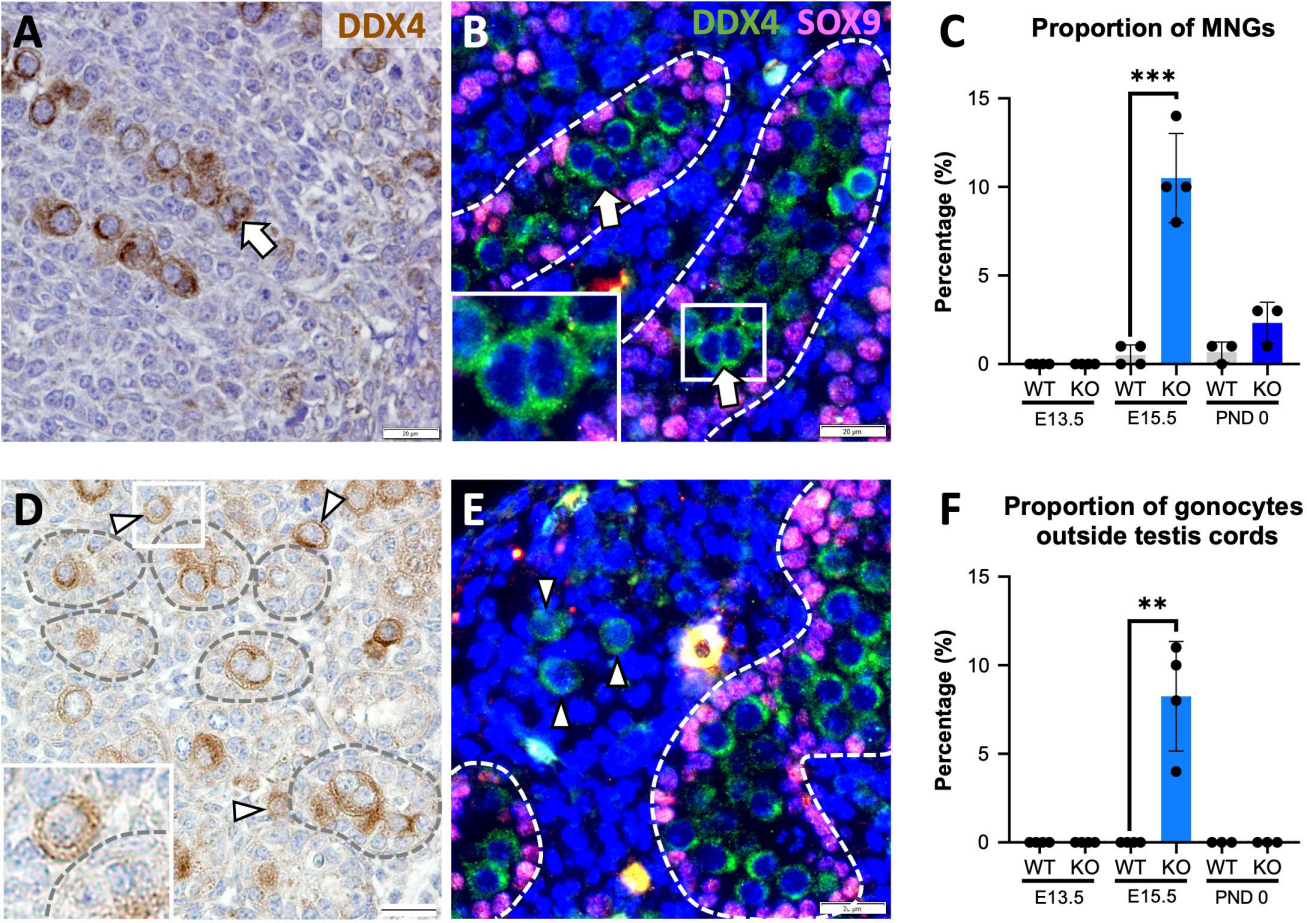
Multinucleated gonocytes, and gonocytes outside the testis cords are characteristics of *Inha* KO fetal testes. Representative images of multinucleated gonocytes (MNGs) (arrow) and gonocytes outside the testis cords (arrowhead) in E15.5 *Inha* KO mouse testes by immunohistochemistry **(A, D)** and indirect immunofluorescence **(B, E)**. Scale bars = 20 µm. Cords outlined by dotted line. Quantification of MNGs **(C)** and proportion of gonocytes outside the testis cords **(F)** in *Inha* WT and KO mouse testes at E13.5, E15.5 and PND 0, n=3-4 animals/age/genotype. Graphs show mean ± SD. Statistical differences were determined using a two tailed unpaired t test, ** p<0.01, *** p<0.001.

Since the absence of activin A results in decreased Sertoli cell and increased gonocyte numbers in newborn (P0) *Inhba* mouse testes (6), we studied the reciprocal condition of unopposed activin A in *Inha* WT and KO testes to learn how these cells were affected. Two surprising gonocyte phenotypes were immediately evident in E15.5 *Inha* KO testes: multinucleated gonocytes (MNGs) (**Figures 1A, B**) and gonocytes outside the cords (**Figures 1D, E**), each of which was only rarely or not observed in wildtype testis sections. The term ‘multinucleated’ refers here to the observation of ≥2 nuclei located within the cytoplasm of a single cell, with bi-nucleated (2 nuclei/cell) germ cells most frequently identified (**Figures 1A** (IHC), **B** (IF)). We quantified the extent of this anomaly at E13.5, directly after sex determination, at E15.5, and at PND 0. Care was taken to discriminate between MNGs and two closely opposed germ cells (see Materials and Methods). Notably, although MNGs were observed in E15.5 KO testes, none were present in E13.5 or PND 0 *Inha* KO testes (**Figure 1C**), highlighting that a potential window of germ cell vulnerability to elevated activin A occurs before E15.5. The proportion of MNGs observed in E15.5 testes significantly increased from 0.5% in WT to ~ 10% in *Inha* KO testes (20-fold increase); by PND 0, the percentage of gonocytes scored as MNGs decreased to 3% which did not differ from WT (p = 0.0890) (**Figure 1C**).

**Figure 2:**
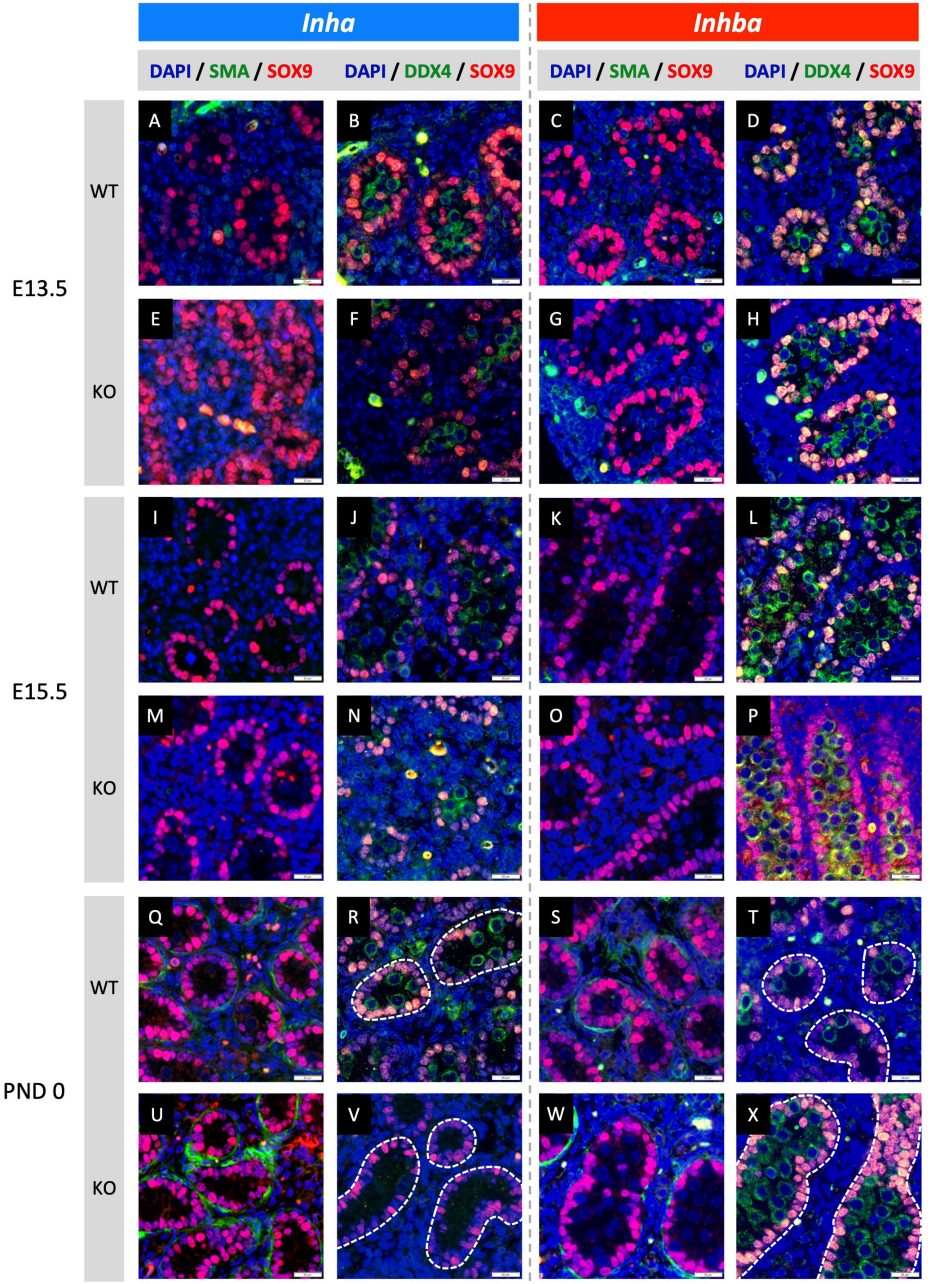
Effects of high and low activin A levels on fetal mouse testis development. Representative images of WT and KO testes from E13.5, E15.5 and PND 0 *Inha* and *Inhba* mouse testes. Immunofluorescent detection of SMA (peritubular myoid cells) and SOX9 (Sertoli cells) images allow comparison of cord formation and Sertoli cell number between WT **(A, C, I, K, Q, S)** and *Inha KO* **(E, M, U)** and *Inhba* KO **(G, O, W)** mouse testes. DDX4 (germ cells) and SOX9 images reveal gonocyte frequency and localisation between WT **(B, D, J, L, R, T)** and *Inha* KO **(F, N, V)** and *Inhba* KO **(H, P, X)** mouse testes. Scale bars = 20 µm. Nuclei were stained with DAPI. Cords outlined with white dotted line.

**Figure 3:**
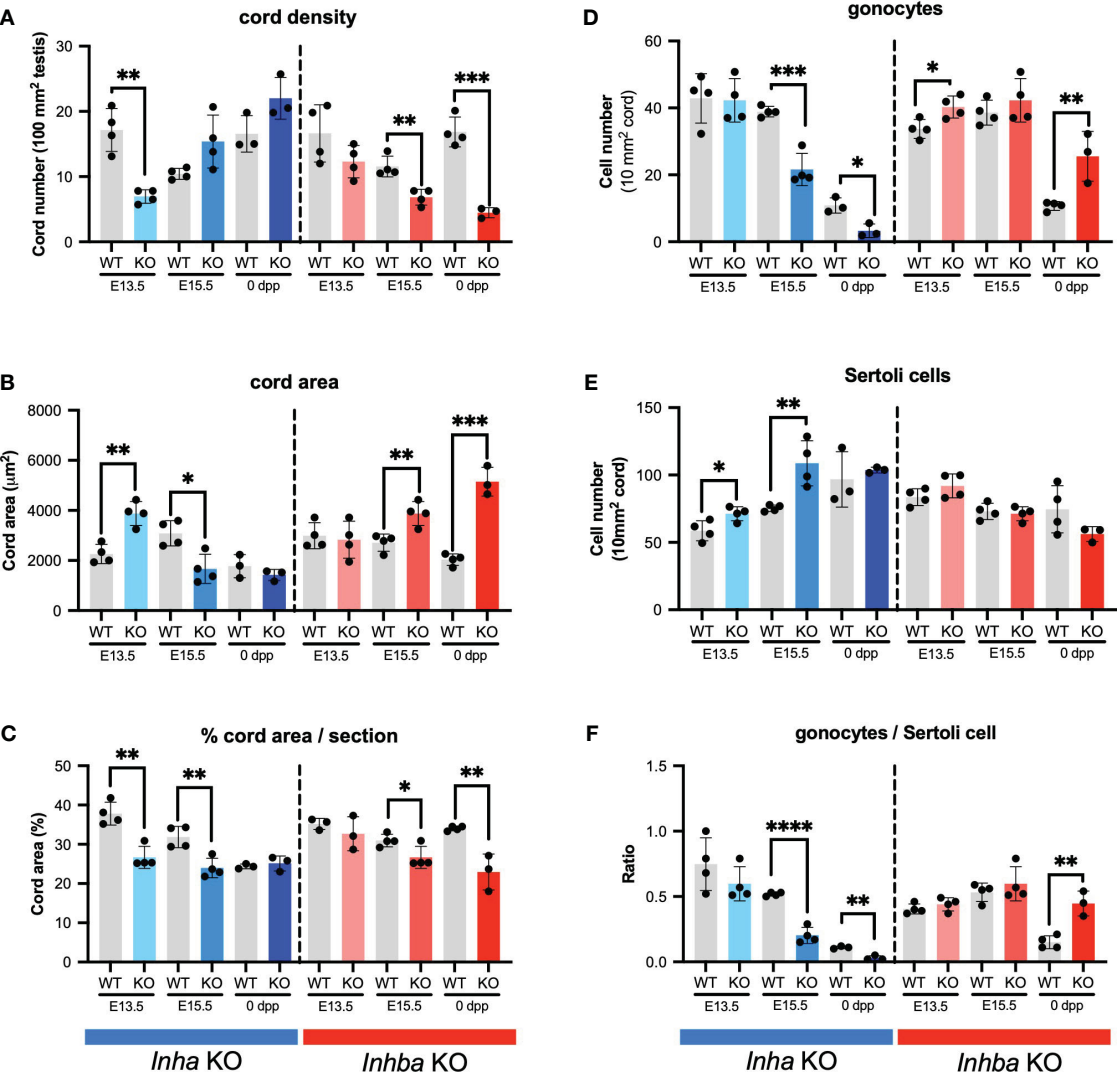
Activin A regulates cord formation, Sertoli cell, and gonocyte cell number in fetal mouse testes. Graphs show **(A)** number of testis cords/100 mm^2^ testis cross section, **(B)** average cord area (µm^2^)/testis cross section, **(C)** % cord area/testis cross section, **(D)** gonocyte number/10mm^2^ testis cord area, **(E)** Sertoli cell number/10mm^2^ testis cord area, **(F)** ratio of gonocyte/Sertoli cells in *Inha* WT and KO (grey/blue bars), *Inhba* WT and KO (grey/red bars) mice. Graphs show mean ± SD, with individual data points from n = 3-4 animals per genotype. Three sections were analysed per animal and statistical differences were determined by two-tailed unpaired t-test, * p<0.05, ** p<0.01, *** p<0.001, **** p<0.0001.

Gonocytes normally become enclosed within Sertoli cells in cords by E13.5, thus their presence outside cords in *Inha* WT and KO testes at E15.5 is abnormal. None were observed outside cords at E13.5, suggesting that cord formation begins normally (**Figure 1F**), however ~8% of *Inha* WT and KO testis germ cells were in the interstitium by E15.5 (**Figure 1F**). At PND 0, no gonocytes were identified outside cords (**Figure 1F**), although the total gonocyte number was markedly reduced (**Figures 2U, V**; **3D**). These data demonstrate how elevated activin A could alter embryonic germline fate and severely reduce the number of gonocytes present at the start of postnatal life.

### Activin A influences germline niche formation in fetal testes

Fetal testis cord development underlies establishment of the germline niche. We next assessed testis cord formation and relative Sertoli and germ cell numbers in fetal mice lacking inhibin (*Inha* KO), comparing these with testes lacking activin (*Inhba* KO) in which effects had been previously identified. Sertoli and germ cells were detected using SOX9 and DDX4, respectively. Testis cord borders were discerned based on SOX9 Sertoli cell nuclear staining at E13.5 and E15.5, and using SMA at PND 0 in *Inha* WT and KO (E13.5 - **Figures 2A, B, E, F**; E15.5 - **Figures 2I, J, M, N**; and PND 0 - **Figures 2Q, R, U, V**) and *Inhba* WT and KO mouse testes (E13.5 - **Figures 2C, D, G, H**; E15.5 - **Figures 2K, L, O, P**; and PND 0 - **Figures 2S, T, W, X**). The impact of activin deficiency or elevated bioactivity was most readily observed in PND 0 testis sections. At this age, germ cell numbers, cord size and the frequency of cord cross sections were markedly different in testes from both *Inha* KO and *Inhba* KO mice (**Figures 2V, X**) compared to WT (**Figures 2R, T**). *Inha* KO testes had fewer gonocytes, while in the reciprocal condition of reduced activin A, the substantial increase in gonocyte abundance inside *Inhba* KO cords correlated with previously published data (6).

These outcomes were quantified by measuring cord parameters relative to inferred activin A levels. In *Inha* KO testes at E13.5, the density of cords in each section was significantly lower compared to WT but did not differ at E15.5 and PND 0 (**Figure 3A**). The average cord area in *Inha* KO testes was significantly higher than in WT at E13.5, lower at E15.5, but comparable to WT at PND 0 (**Figure 3B**). The percentage of each testis cross section occupied by cords was significantly lower at E13.5 and E15.5 relative to WT, but not different at PND 0 (**Figure 3C**). These data suggest that elevated activin A bioactivity disrupts initial processes relating to fetal testis cord development, however by birth the overall cord dimensions are normal. The impact of reduced levels of activin A is evident at later time points compared to in *Inha* testes. In *Inhba* KO testes at E15.5 and PND 0, cord number was significantly lower (**Figure 3A**), the average cord area was higher (**Figure 3B**), and the percentage of testis area occupied by cords was significantly lower (**Figure 3C**). These data are consistent with previous reports showing the absence of activin A progressively leads to a smaller number of cords with an increased cord diameter (6, 12, 46).

We investigated whether these cord phenotypes reflect an impact of activin A on the relative density of germ and Sertoli cells. In *Inha* KO mice, the density of gonocytes per cord area is reduced at E15.5 and PND 0 (**Figure 3D**), while Sertoli cell density is greater in E13.5 and E15.5 testes (**Figure 3E**). The higher Sertoli cell density at the two earlier ages suggests that Sertoli cell population expansion is advanced during early fetal life in the *Inha* KO mice. *Inhba* KO testes had more gonocytes (**Figure 3D**) as previously (6), while in contrast, Sertoli cell density was not significantly different from in WT testes at any age; at PND 0 it was reduced, but not significantly (p = 0.1474) (**Figure 3E**). This latter finding differs from the significant dose-dependent decrease in Sertoli cell number at PND 0 reported previously (6); our previous application of the optical disector method, suited to counting irregular-shaped nuclei such as those of Sertoli cells, is likely to have led to this discrepancy.

Assessment of gonocyte/Sertoli cell ratios provided a clear measure of the physiological importance of activin A on the relationship between Sertoli and germ cells. Throughout development, Sertoli cells are always in close contact with germ cells, producing factors essential to their support and maturation, and each Sertoli cell supports a finite number of spermatogenic cells (47). The gonocyte/Sertoli cell ratio was significantly and progressively reduced in *Inha* KO testes at E15.5 and PND 0 and was significantly higher in *Inhba* KO testes at PND 0 (**Figure 3F**). These data provide evidence that activin A bioactivity is an important contributor to establishing the niche that determines the spermatogenic potential of the newborn testis.

### RNAseq reveals targets of elevated activin A in the testis

**Figure 4:**
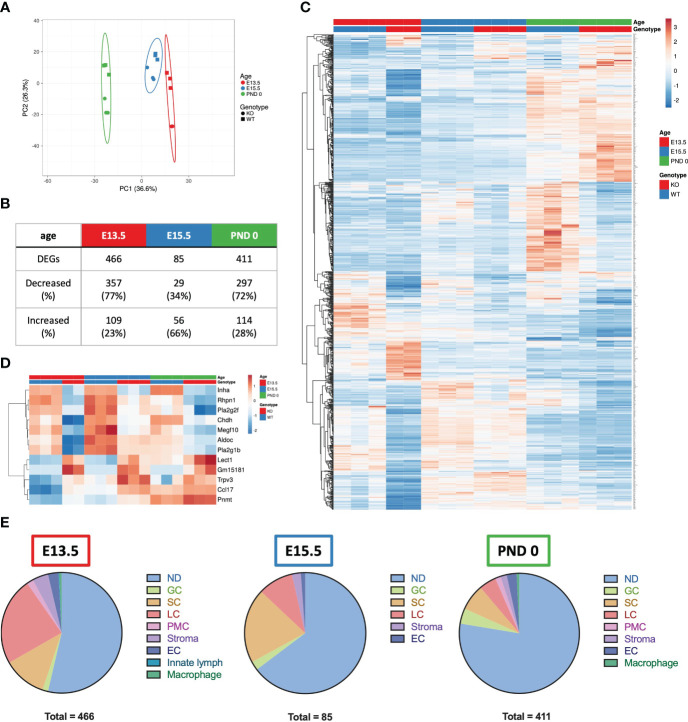
Summary of E13.5, E15.5 and PND 0 *Inha* WT and KO whole mouse testes RNAseq analyses outcomes. **(A)** Principle component analysis plot of the differentially expressed genes (DEGs) in E13.5, E15.5 and PND 0 whole mouse testes. Unit variance scaling is applied to rows; SVD with imputation is used to calculate principal components. X and Y axis show principal component 1 and principal component 2 that explain 36.6% and 26.3% of the total variance, respectively. Prediction ellipses are such that with probability 0.95, a new observation from the same group will fall inside the ellipse. N = 17 data points. **(B)** Table showing the total number of DEGs, and number and percentage (%) of DEGs increased and decreased at each age. DEGs were identified using DEGUST (FDR < 0.05 and absolute fold change ≥ 1.5). **(C)** Heatmap showing 876 DEGs identified in E13.5, E15.5 and PND 0 *Inha* WT and KO samples. **(D)** Heatmap showing 12 DEGs that were altered at all ages. PCA plot and heatmaps were generated using ClustVis. For both heatmaps, rows are centred; unit variance scaling is applied to rows. Rows are clustered using correlation distance and average linkage. **(E)** Pie charts showing the predominant cellular localisation of the DEGs identified at each age, where possible. Localisation determined using single cell RNAseq data (48). ND, not determined; GC, germ cells; SC, Sertoli cells; LC, Leydig cells; PMC, peritubular myoid cells; Stroma, stromal cells; EC, endothelial cells; Innate lymph and Macrophages.

To identify the transcriptional differences in the fetal testis arising from unopposed activin A, whole testis RNA sequencing of *Inha* WT and KO testes was performed. Principle component analysis (**Figure 4A**) illustrated age and genotype differences. Four hundred and sixty-six differentially expressed genes (DEGs) were identified between *Inha* KO and WT testes at E13.5, 85 at E15.5 and 411 at PND 0 (**Figure 4B**) using an FDR < 0.05 and absolute fold-change ≥ 1.5. A heat map illustrates transcript cohorts that are differentially affected by the absence of inhibin at each age (**Figure 4C**). Amongst the DEGs, only 12 were significantly altered at all three ages including *Inha* (**Figure 4D**). Transcripts from 7 genes were decreased (*Inha*, *Rhpn1*, *Pla2g2f*, *Chdh*, *Megf10*, *Aldoc*, *Pla2g1b*), and 5 were increased (*Lect1*, *Gm15181*, *Trpv3*, *Ccl17*, *Pnmt*).

To determine whether one particular cell type was affected in the *Inha* KO testes, the cell-type expression of DEGs was investigated by interrogating a single cell (sc)-RNAseq dataset obtained from E18.5, PND 2 and PND 7 mouse testes (48). Most DEGs were not cell-specific (present in more than one cell type, indicated as ‘not determined’ (ND), **Figure 4E**). The remaining transcripts were predominantly expressed in a single cell type (**Figure 4E**): germ cells, Sertoli cells, Leydig cells, peritubular myoid cells, endothelial cells, stromal cells, and innate lymph cells or macrophages. The deduced cellular localisation for all DEGs are listed in **Supplementary Table 1**. At E13.5, the majority of DEGs were present in Leydig cells (23.6%), followed by Sertoli cells (11.6%). By E15.5, this pattern was reversed (Sertoli (20%) and Leydig cells (9.4%), and by PND 0 there was a bias towards cell-specific DEGs being expressed in Leydig (4.6%), Sertoli (7.1%) and germ cells (4.1%) (**Figure 4E**).

**Figure 5:**
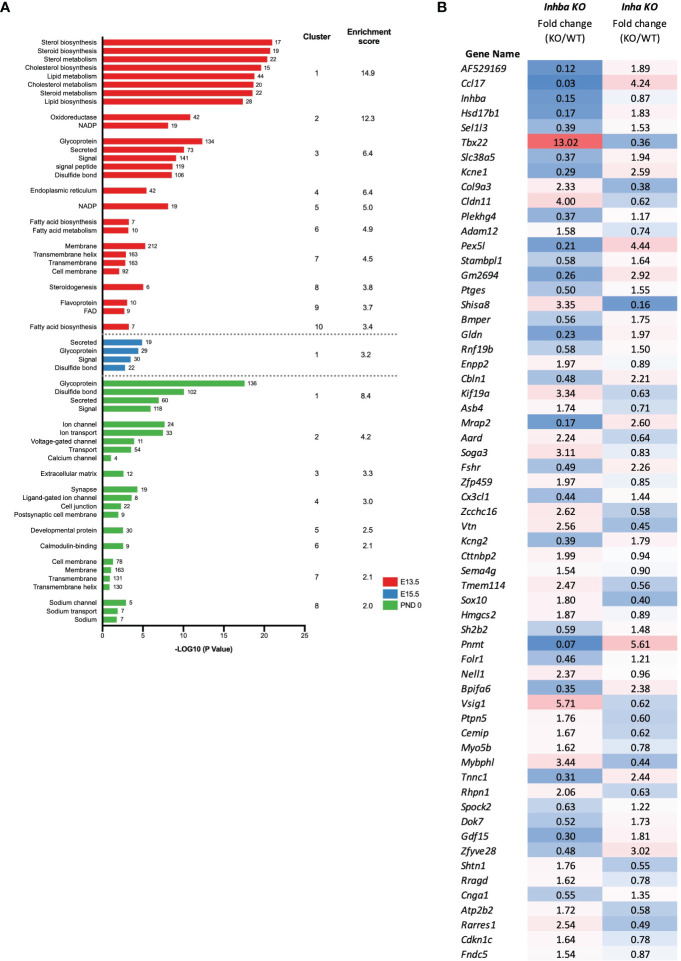
Identification of biological processes affected in *Inha* KO embryonic testes. **(A)** Functional annotation analysis of the DEGs identified at individual ages (E13.5; red, E15.5; blue, PND 0; green). Graph shows associated UniProt keywords and significance (-Log10 (P value)), top clusters and enrichment scores identified using DAVID analysis. Numbers at the end of each bar represent the number of genes associated with each keyword. **(B)** Signature of activin A bioactivity in the fetal mouse testis. List of 61 common DEGs identified in *Inhba* KO and *Inha* KO RNAseq datasets showing reciprocal expression. Fold change (based on average C.P.M.) in E15.5 *Inhba* and *Inha* KO samples is shown, with blue shading indicating a decrease and red shading an increase in the KO compared with WT.

The DAVID functional clustering tool was applied to the DEGs in *Inha* KO testes at each age to investigate the potential cellular consequences of excess activin A bioactivity. At E13.5, the DEGs were significantly enriched in functional terms relating to the metabolism and synthesis of sterols, steroids, and lipids (**Figure 5A**). At E15.5, the DEGs were associated with a single functional cluster annotated as secreted proteins and glycoproteins (**Figure 5A**). At PND 0, the top 8 most significant clusters were enriched for terms such as glycoproteins, secreted proteins, ion transport, extracellular matrix and cell junctions (**Figure 5A**).

To identify genes that could be directly responsive to activin A in embryonic testes, we compared all DEGs in the *Inha* KO whole testes (this study) with DEGs identified in the *Inhba* KO E15.5 somatic cells (19). In total, 61 transcripts were altered in both datasets (**Figure 5B**) and exhibited reciprocal changes in testes from mice with high and low activin A levels (**Figure 5B**). While these genes provide a signature of altered activin A bioactivity in the fetal testis, we propose that they are strong candidates for being direct targets of activin signalling.

### Steroidogenesis is affected in Inha KO fetal mouse testes

**Figure 6:**
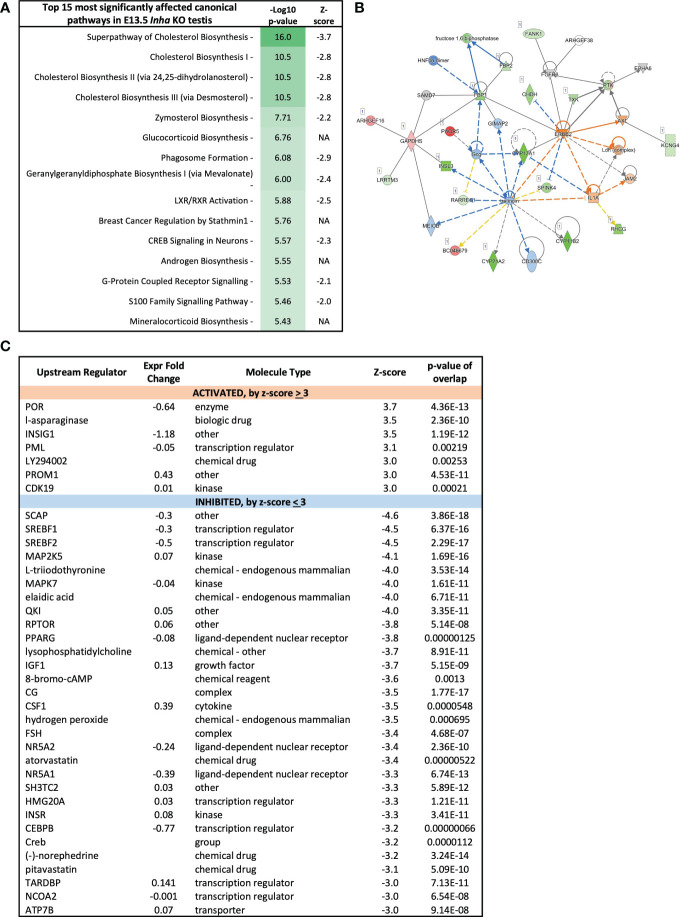
Cholesterol biosynthesis pathways inhibited in E13.5 *Inha* KO mouse testes, identified using Ingenuity Pathway Analysis (IPA). **(A)** Top 15 most affected canonical signalling pathways. Table shows pathway name, - log10 p-values, and z-score indicative of pathway activation (Z-score ≥ 3) or inhibition (Z-score ≤ −3); NA indicates z-score values > -2 and < 2. **(B)** Lipid metabolism network showing differentially expressed transcripts in E13.5 *Inha* KO testes. Upregulated transcripts are shaded red (known) or orange (predicted). Downregulated transcripts are shaded green (known) or blue (predicted). Colour intensity indicates the degree of up or downregulation. Solid lines indicates that a direct interaction between two gene products has been detected and a dotted line means an indirect interaction is supported. Relationships inconsistent with published reports are shown with yellow lines. **(C)** Predicted upstream regulators of genes differentially expressed in E13.5 *Inha* KO testes with Z score cut off of ±3.0 identified using Ingenuity Pathway Analysis. Z-score ≤ -3 indicates inhibition and a Z-score ≥3 indicates activation. Expression fold-change reflects WT versus KO values. P-value of overlap indicates a statistically significant overlap between dataset genes and genes that are regulated by an upstream regulator.

Because altered levels of transcripts important for lipid and steroid metabolism are key outcomes of unopposed activin A in E13.5 *Inha* KO testes, Ingenuity Pathway Analysis was employed to evaluate the broader implications on this and other signalling and cellular activities. The top 15 cellular pathways affected in the E13.5 testes are identified (**Figure 6A**), and 11 of these (73%) are predicted to be inhibited (a z-score < -2). The top 4-most significantly affected pathways relate to cholesterol biosynthesis, while others relate to synthesis of zymosterol, glycocortioids and mineralcorticoids and the LXR/RXR, GPRS, CREB and S-100 family signalling pathways (**Figure 6A**). Network analysis (**Figure 6B**) indicates robust downregulation of genes involved in lipid and steroid metabolism, including *Insl3*, *Cyp17a1, Cyp11b2, Cype1a2*, amongst other transcripts expressed exclusively in fetal testis Leydig cells, including *Crhr* and *Mc2r* (49). These results demonstrate that emergence of steroidogenic Leydig cells, in either number and/or function, is disrupted in E13.5 *Inha* KO testes. Ingenuity Pathway Analysis identified candidate upstream regulators that influence the levels of DEGs in E13.5 *Inha* KO testes (**Figure 6C**). Based on z-scores, there was a strong bias towards inhibitory factors, with 7 activating and 34 inhibitory (90%) (**Figure 6C**, **Supplementary Table**; z-score of < -3 and > 3). **Supplementary Table 2** contains the complete list of potential upstream regulators, their activation status and target genes.

**Figure 7:**
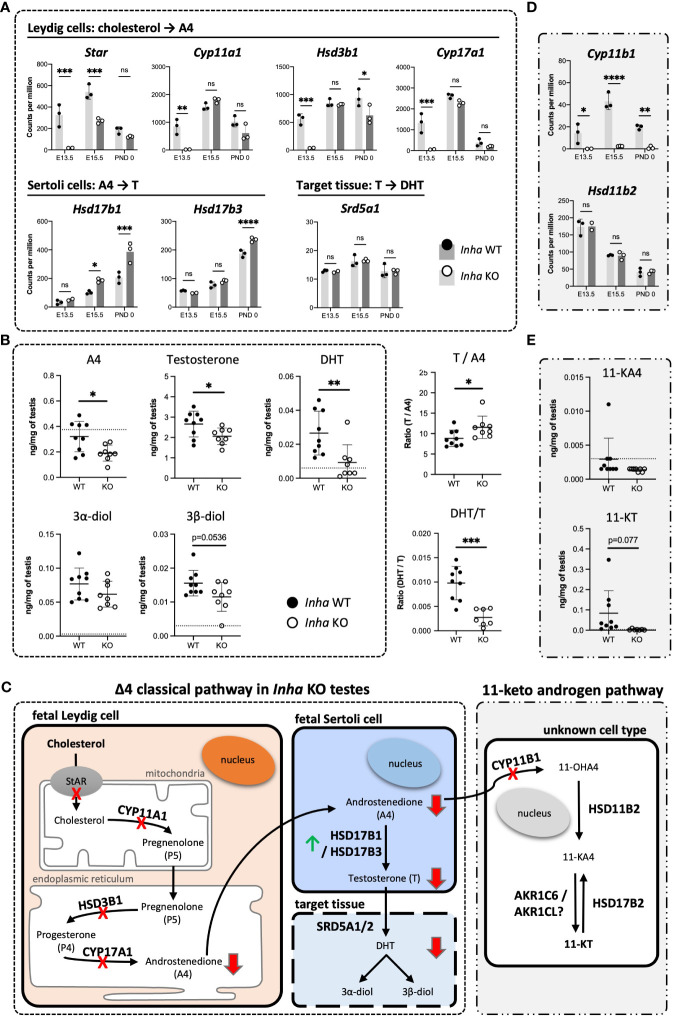
Impaired testosterone and DHT synthesis in fetal mouse testes with elevated activin A **(A)** Transcripts encoding enzymes that convert cholesterol to A4 in fetal Leydig cells (*Star, Cyp11a1, Hsd3b1, Cyp17a1*), androstenedione (A4) to testosterone (T) (*Hsd17b1, Hsd17b3*) in Sertoli cells, and T to DHT (*Srd5a1*). **(B)** Transcripts involved in conversion of A4 to 11-KT (*Cyp11b1, Hsd11b2, Hsd17b2*). Graphs show relative expression measured by Fluidigm in *Inha* WT (black symbol) compared with *Inha* KO (white symbol) whole gonads aged E13.5, E15.5 and PND 0. Values represent mean ± SD. Significance determined using an unpaired two-tailed t-test, ns, not significant, *P < 0.05, **P < 0.01, ***P < 0.001, ****P < 0.0001. **(C, D)** Hormone measurements in E17.5 *Inha* x OG2 mouse testes. Graphs show ng/mg of testis measured by LC-MS in *Inha* x OG2 WT (black circles) and KO (white circles) E17.5 paired testes. N = 8-9/genotype. Mean ± SD. DL, detection limit set at: limit of detection (LOD) per analyte (dotted line). Not detectable values set at: LOD per analyte divided by 2. **(E)** Summary schematic of the Δ4 classic steroidogenic and the 11 keto-androgen pathway in fetal mouse testes. In fetal Leydig cells, StAR (steroidogenic acute regulatory protein) binds and transfers cholesterol from the outer to the inner mitochondrial membrane where the enzyme CYP11A1 catalyzes conversion to pregnenolone (P5). Thereafter, P5 is transferred to the endoplasmic reticulum, where HSD3B1 and CYP17A1 enzymes convert it to progesterone (P4) and androstenedione (A4), respectively. The final step in testosterone synthesis occurs in the fetal Sertoli cells and involves the reduction of A4 to T, catalyzed by HSD17B3 and HSD17B1. T is reduced to form dihydrotestosterone (DHT), and then 3α-diol and 3β-diol in target tissues. A lack of HSD17b1 and HSD17b3 enzymes can result in A4 being diverted into the 11-ketoandrogen pathway. Red crosses and red arrows indicate reduced transcripts and hormones respectively. Green arrow indicates elevated transcripts. 11-OHA4, 11β-hydroxyandrostenedione; 11-KA4, 11-keto androstenedione; 11-KT, 11-ketotestosterone; DL, decision limit; E, embryonic day; KO, knockout; LOD, limit of detection; SD, standard deviation; WT, wild type.

These analyses indicate that the most affected cellular processes in E13.5 *Inha* KO testes relate to Leydig cell function (**Figure 4E**) and impact on steroid/lipid biosynthesis and metabolism (**Figures 5A**, **6A**). Levels of key steroidogenic enzyme transcripts involved in the Δ4 classical steroidogenesis pathway, the predominant pathway in rodents, are presented in **Figure 7**. At E13.5, transcripts produced in fetal Leydig cells encoding *Star*, *Cyp11a1*, *Hsd3b1* and *Cyp17a1* were robustly detected in WT testes but were barely detected in *Inha* KO (**Figure 7A**). In contrast, there were no differences in transcripts encoding steroidogenic enzymes *Hsd17b1*, *Hsd17b3* and *Srd5a1*, which are exclusively expressed in fetal Sertoli cells at this age (**Figure 7A**). In *Inha* KO testes at E15.5, most of steroidogenic transcripts were unaffected except for *Star* (reduced) and *Hsd17b1* (increased). At PND 0, steroidogenic enzyme transcripts produced in Leydig cells were not significantly different except for *Hsd3b1* (decreased) whereas the activin A-responsive steroidogenic enzymes expressed in Sertoli cells, *Hsd17b1* and *Hsd17b3* (19, 48), were significantly increased.

To assess the outcomes of these transcriptional differences in *Inha* KO testes, several steroids were measured in E17.5 mouse testes using mass-spectrometry. Androstenedione (A4), testosterone and DHT were each significantly lower in *Inha* KO testes compared to their WT counterparts (**Figure 7B**), indicating that the overall level of steroidogenesis was reduced. The ratios of particular steroids highlighted key affected steroid biosynthesis steps (**Figure 7B**): the elevated T/A4 ratio in *Inha* KO testes was consistent with increased Sertoli cell expression of testosterone synthesising enzymes *Hsd17b1* and *3* (**Figure 7A**), while the lower DHT/T ratio indicates 5alpha-reduction of testosterone is diminished. These results provide evidence that elevated activin A transiently impacts on Leydig cell gene transcription (**Figure 4E**), reducing the production of androstenedione (A4) (**Figure 7C**) at E13.5, whereas at later time points, the capacity for Sertoli cells to convert A4 into testosterone (T) is elevated.

We previously identified that levels of the 11-keto steroids 11-KA4 and 11-KT are increased when activin A are decreased (in *Inhba* KO testes) at E17.5 (19), suggesting their synthesis was responsive to activin A bioactivity. During the conversion of A4 to the 11-ketoandrogens, CYP11B1 converts A4 to the first 11-keto derivative, 11-OHK4 (**Figure 7C**). 11-OHK4 is then converted to 11-KA4 by HSD11B2, and 11-KA4 is converted to 11-KT by AKR1C6/AKR1CL (**Figure 7C**). Importantly, *Cyp11b1* was barely detected in *Inha* KO testes at all ages but *Hsd11b2* levels were not altered (**Figure 7D**), and *Hsd17b2* was not detected (data not shown). We were unable to confidently measure 11-KA4 in E17.5 testes however 11-KT, measurable in WT testes, was undetectable in *Inha* KO testes (**Figure 7E**) indicative of reduced 11-keto androgen production in testes with unopposed activin. Overall, these data suggest that elevated activin A levels in early fetal life have a major impact on testicular steroidogenesis, affecting first fetal Leydig cells, then Sertoli cells (**Figure 7A**). The overall outcome is that levels of both testosterone and DHT, androgens crucial to testicular development and fetal masculinisation are reduced.

### Activin A and MEHP exposures result in similar germ cell phenotypes and can regulate common transcripts

**Figure 8:**
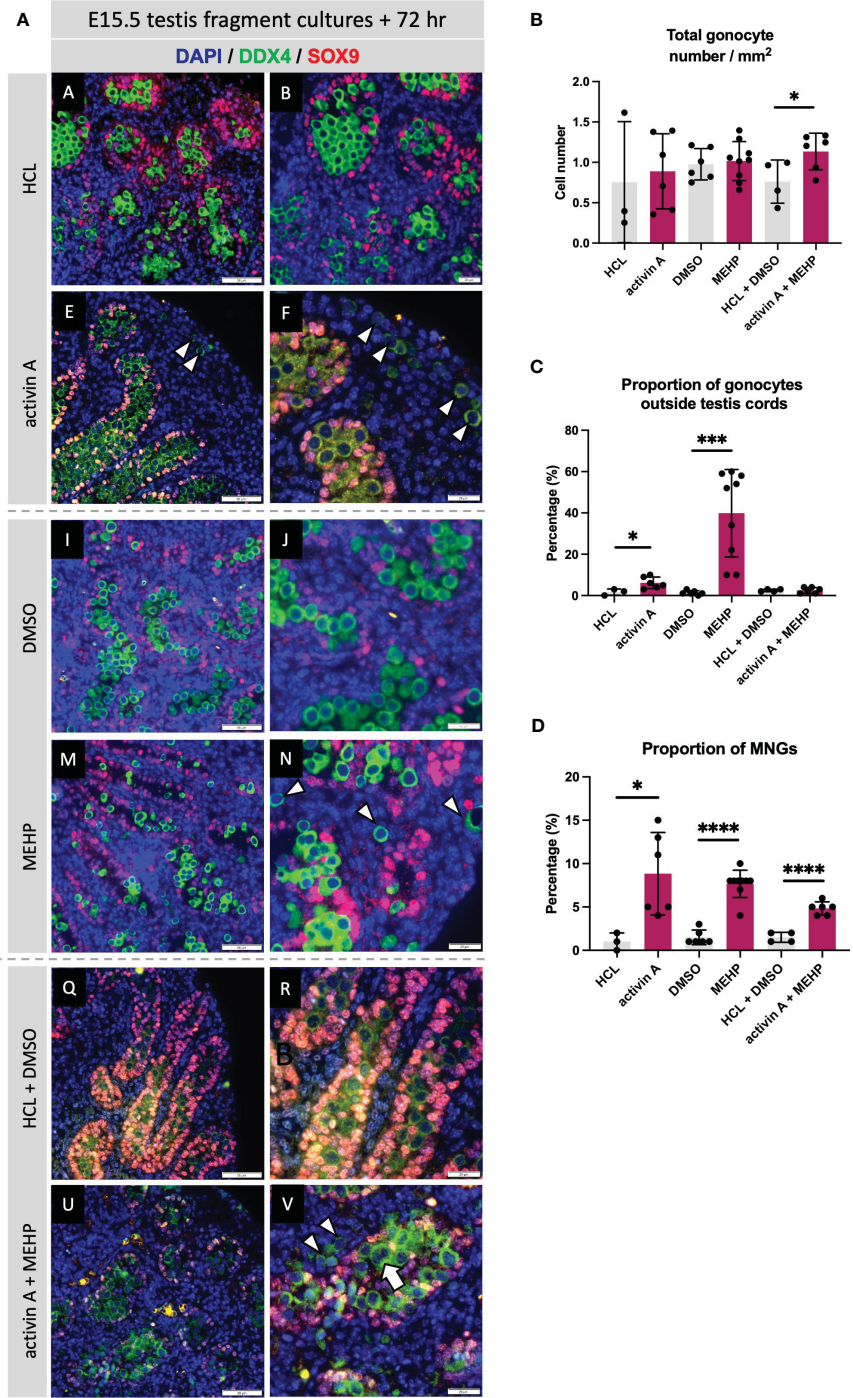
Acute exposure to activin A and MEHP increases the proportion of multinucleated gonocytes and gonocytes outside the cords. **(A)** Representative images of E15.5 WT testis fragment cultured with activin A, MEHP, activin A and MEHP combined, or appropriate vehicle controls (HCL for activin A, DMSO for MEHP) for 72 hrs. Immunofluorescent detection of germ (DDX4) and Sertoli cells (SOX9) highlight presence of gonocytes outside the cords and multinucleated gonocytes. Scale bars = 20 µm. All images stained with DAPI. Graphs show **(B)** Total gonocyte number/mm^2^, **(C)** Proportion of gonocytes outside testis cords, **(D)** Proportion of multinucleated gonocytes (MNGs) in E15.5 testis fragments treated with appropriate controls (grey bars), activin A, MEHP or activin A + MEHP combined (purple bars) for 72 hrs. Graphs show mean ± SD, along with individual data points. N = 3 animals per genotype. Statistical differences were determined by two-tailed unpaired t-test, * p<0.05, *** p<0.001, **** p<0.0001.

The absence of activin A (in *Inhba* KO testes) has been reported to predominantly affect somatic cells, in particular the Sertoli cells (12, 19), whilst MEHP predominantly impacts Leydig cell functions in rodents (27, 50). Nevertheless, due to the common germ cell phenotypes of increased MNG numbers and gonocytes present in the interstitium at E15.5 following MEHP exposure (27) and reported here for *Inha* KO testes, we hypothesised that activin and MEHP regulate common pathways and genes. E15.5 mouse testes fragments were cultured for 72 hrs with activin A (25 ng/ml), MEHP (200 µm) or both factors. Immunofluorescence images showed that culture with activin A and MEHP, individually or combined, resulted in increased frequency of MNGs within cords and gonocytes outside cords (**Figure 8A**). Quantification showed neither activin A or MEHP alone altered the total density of gonocytes, however there was a modest but statistically significant increase in cultures with both factors present (**Figure 8B**). The proportion of gonocytes outside cords was increased by activin A and robustly enhanced by MEHP (**Figure 8C**). Interestingly, testis fragments exposed to both activin A and MEHP did not have a higher proportion of gonocytes outside cords (**Figure 8C**). Fragments cultured with activin A or MEHP, or both combined, displayed a striking increase in the proportion of multinucleated gonocytes (MNGs) (**Figure 8D**).

To test for relationships in transcriptional regulation by activin and MEHP, transcript levels measured in whole fetal testes with different activin bioactivity levels were compared with those in E15.5 testis fragments cultured for three days with MEHP. Known activin-responsive transcripts (in *Inhba* testes; (19)) that were also identified as DEGs in whole *Inha* KO vs WT testes (this study), and selected germ cell markers, including markers of spermatogonial stem cells (SSCs), were examined.

**Figure 9:**
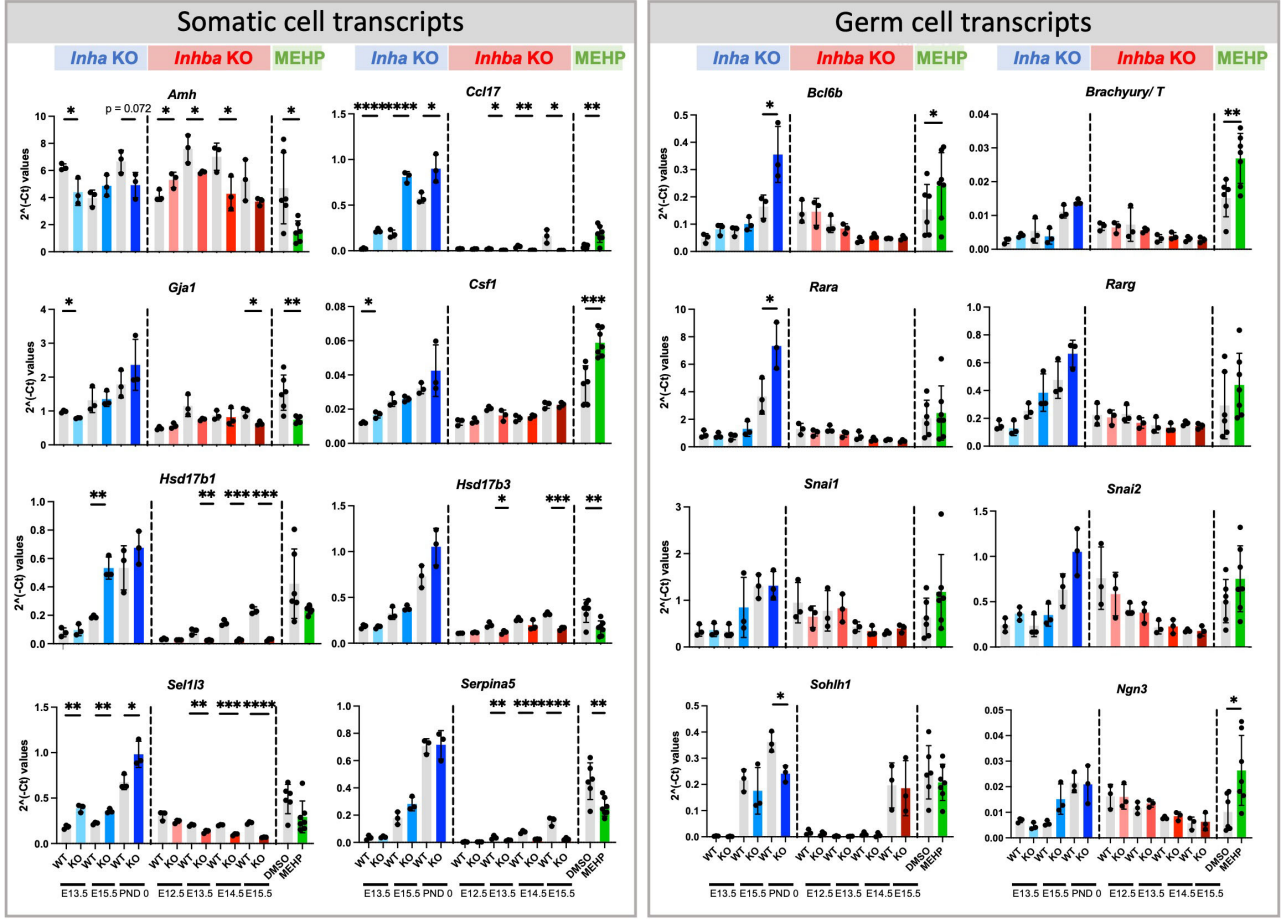
Somatic and germ cell transcripts in whole mouse testes commonly affected by exposure to chronically altered activin A and acute exposure to MEHP. Fluidigm analysis of *Inha* WT and KO (grey/blue bars), *Inhba* WT and KO (grey/red bars) whole testes and of testis fragments cultured with DMSO (vehicle control) or MEHP for 72 hrs (grey/green bars). Graphs show ΔΔCt normalised to the average of housekeeper genes (*Canx* and *Mapk1*) measured in independent whole gonads aged E12.5 – PND 0. N=3/age/genotype or n=6-7/culture condition. Gonocyte transcripts were further normalized to *Ddx4* to account for germ cell population changes. Values represent mean ± SD. Significance determined using two-tailed unpaired t-test, * p<0.05, ** p<0.01, *** p<0.001, and **** P<0.0001.

**Figure 10:**
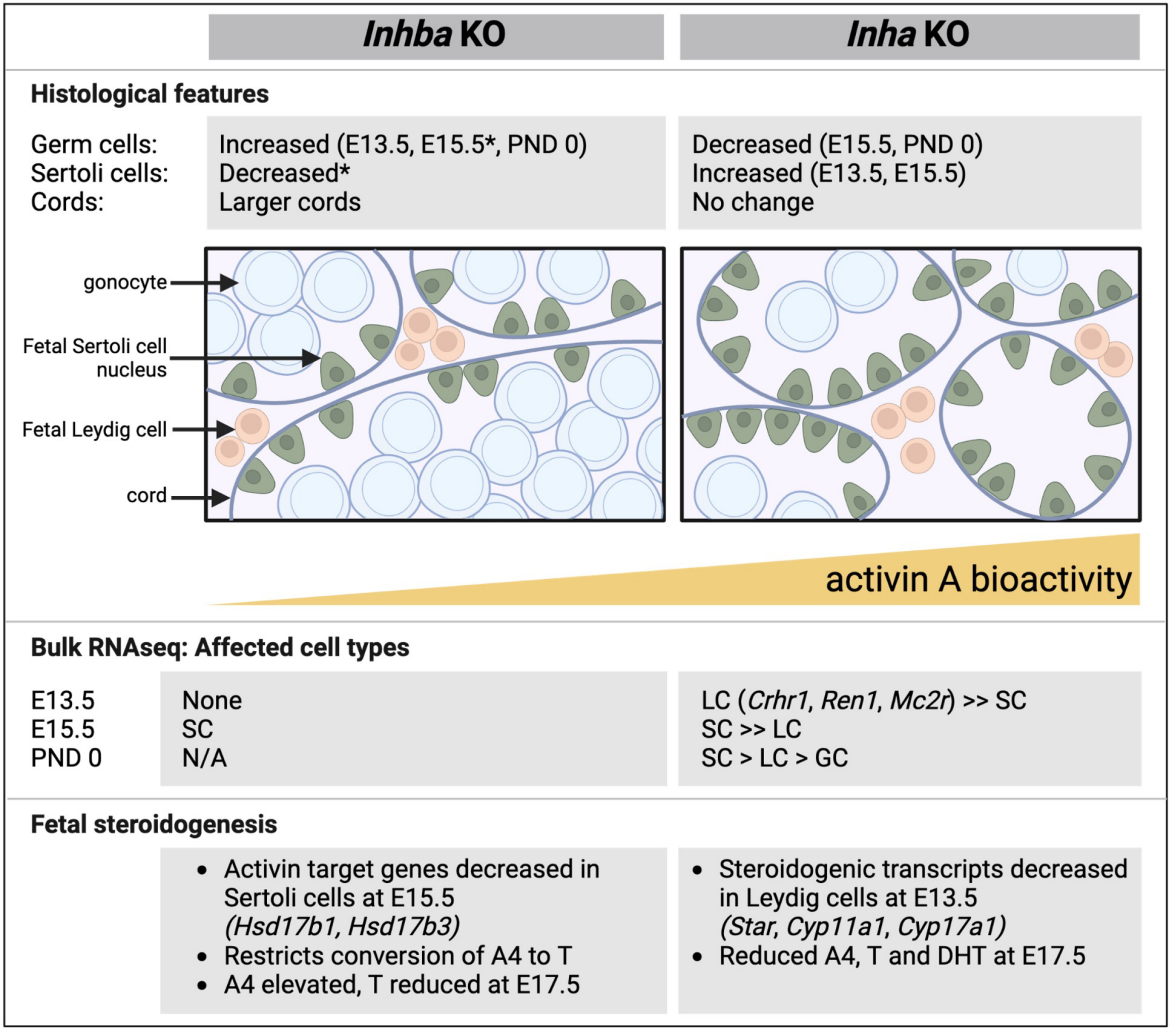
Comparative summary of fetal testis phenotypes observed in mice lacking activin A (I*nhba* KO) and with unopposed activin A (*Inha* KO). Key histological features, outcomes from RNA sequencing, and features relating to steroid production shown in affected cell types. In general, there is a reciprocal relationship between germ cell and Sertoli cell numbers. In the *Inhba* KO testes, Sertoli cells are the predominantly affected cell type, evident at E15.5, while in *Inha* KO testes, Leydig cells are affected at E13.5. In both models, T production is reduced. Data are collated from this manuscript and previous studies (6, 12, 19). E, embryonic; PND, postnatal day; SC, Sertoli cell; LC, Leydig cells; GC, germ cells; A4, androstenedione; T, testosterone; DHT, dihydrotestosterone, N/A, not available; * indicates data from 6.

Amongst the activin-responsive somatic cell transcripts, several that are expressed in fetal and immature Sertoli cells were identified as significantly altered in all three groups (**Figure 9**). *Amh*, encoding Anti-Mullerian Hormone, was significantly decreased at multiple ages in both *Inha* and *Inhba* mouse testes and in MEHP cultures, while in contrast, levels of *Ccl17*, encoding the chemokine CCL17, were positively regulated by activin A (i.e. increased in *Inha* KO and decreased in *Inhba* KO testes). *Ccl17* was also significantly increased by MEHP (**Figure 9**). *Gja1* was significantly lower in E13.5 *Inha* KO testes, E15.5 *Inhba* KO testes and in MEHP-treated samples (**Figure 10**). Also known as *Cx43*, *Gja1* encodes an activin A-regulated gap junction protein, GJA1, crucial for postnatal blood-testis barrier formation and important for Sertoli cell maturation that is affected by *in vivo* DEHP exposure (51). In fetal mouse testes lacking activin A (*Inhba* KO), the Sertoli cell-expressed enzymes Hsd17b1 and Hsd17b3, which convert A4 to T, are reduced while in the contrasting condition of unopposed activin A (*Inha* KO testes), only *Hsd17b1* was higher at E15.5. *Hsd17b1* was not altered by MEHP exposure, however *Hsd17b3* was significantly reduced. *Sel1l3*, which encodes a protein of unknown function and poorly characterised distribution, and *Serpina5*, encoding a serine proteinase inhibitor produced in Sertoli and other cell types, were both increased in testes with elevated activin A (*Inha* KO), decreased in the absence of activin A (*Inhba* KO), and reduced following MEHP treatment. The *Csf1* transcript, encoding cytokine colony stimulating factor 1 (CSF1), is more broadly expressed, being produced in interstitial cells including the vasculature, macrophages, Leydig and peritubular myoid cells (reviewed in (52, 53)). *Csf1* was increased significantly in MEHP-treated testis fragments but increased only modestly at E13.5 in *Inha* KO testes (**Figure 9**). Overall, these findings indicate that the dynamic range of activin A signalling differs between target genes and that many activin-regulated transcripts of functional importance to fetal testis development that are synthesized in Sertoli cells can be altered by exposure to MEHP.

To account for the reduction in gonocytes, measures of germ cell transcripts were normalised to the germ cell marker, *Ddx4*. Amongst the cohort of SSC-specific transcripts examined, regulation by activin was limited to the PND 0 *Inha* KO testis, with *Bcl6b* and *Rara* significantly elevated and *Sohlh1* significantly decreased (**Figure 9**). Exposure to MEHP elevated *Bcl6b*, *T*, and *Ngn3*, each of which normally increase normally between E15.5 and PND 0, which suggests MEHP actions can advance the trajectory of germ cell development.

## Discussion

Activin A is a ubiquitous, highly conserved protein that drives formation of gonadal stromal cell tumours in adult mice which lack its potent inhibitor, inhibin (*Inha* KO) (31). To examine activin A functions in fetal testis biology, mouse models lacking or designed to conditionally ablate activin A (*Inhba* KO, Amhr2^cre/+^; *Inhba^fl^* ^/−^ conditional KO) have been employed (6, 12, 19). Sertoli cells have been established as a key target, with cord formation, germ cell maturation and steroid production all shown to be altered by activin A deficiency. However, during human pregnancy, maternal serum activin A can be elevated in conditions such as pre-eclampsia, fetal growth restriction, infection and exposure to certain medications (2–5). Because activin A can cross the placenta, its potential to influence fetal development should be assessed to understand how exposure to supraphysiological levels of activin A may affect organ development and ultimately adult health. This study has identified for the first time the effects of unopposed/elevated activin A signalling on fetal testis development in the *Inha* KO mouse.

Precursors to Sertoli cells have been identified near the coelomic epithelium in the fetal mouse as early as E10.5; these are *Wnt4+* and *Sox9+* cells that are initially sexually bipotential (termed supporting-like cells, or SLCs) but acquire a sex-specific identity by E12.5 (10). A progenitor *Wnt5*a+ cell population emerging from both coelomic epithelium and mesonephros gives rise to steroidogenic cells that form fetal and adult Leydig cells and peritubular myoid cells, detected from E11.5 onwards (9, 49). While the absence of activin A predominantly affects Sertoli cells at E15.5 (19), our new data reveal that, in the reciprocal condition of elevated activin A bioactivity, fetal Leydig cells are the principal cell type affected at E13.5. The gross reduction of the vast majority of Leydig cell-specific transcripts (49) indicates that this cell population has not emerged appropriately from its precursor population, which normally expands from 0.1% of the total testis population at E12.5 to 4.1% at E13.5 (17). Although *Inha* KO Leydig cells appeared grossly normal at E13.5, it is interesting that adult *Inha* KO testes have fewer Leydig cells (31); of relevance is the demonstration that fetal Leydig cells contribute to the adult population (17).

In the fetal testis, Leydig cells perform the important role of converting of cholesterol to A4, while enzymes in Sertoli cells convert A4 to T. Transcripts encoding several key steroidogenic enzymes (*Star*, *Cyp11a1*, *Hsd3b1*, *Cyp17a1*) were barely detectable at E13.5 in *Inha* KO testes, but the levels of each of these recovered by E15.5, with only *Star* significantly lower than in WT at this age. The observation from Ingenuity Pathway Analyses that several cholesterol biosynthesis pathways were inhibited at E13.5, highlights the profound impact on Leydig cells and/or processes that underpin steroid production. Despite the transient reduction of these transcripts, we recorded a long-term effect on steroid production at E17.5, with reduced A4, T and DHT in *Inha* KO testes, consistent with reduced bioavailability of the enzymes required to produce them. Crucially, this shows the Leydig cells are acutely sensitive to the condition of elevated activin A bioactivity at and immediately after the time of sex determination, with the potential to restrict androgen production at later ages resulting from early exposure. However, the model we used represents a condition of chronic elevation, and the transition from Leydig cell to Sertoli cell transcriptional changes does highlight the functional interdependency of these two cells which are both required for androgen synthesis in the fetal testis. The potential for extra-testicular sources of activin A to regulate these important transitions remains to be explored.

In *Inha* KO mice lacking inhibin proteins, germ cells at E13.5 appear grossly normal and are fully enclosed within the testis cords. However, the reduction in germ cells to 50% of WT levels at E15.5 and PND 0 indicates their vulnerability to elevated activin A bioactivity during early fetal testis development. The formation of multinucleated cells within the nascent cords, and the abnormal appearance of germ cells outside of the cords at E15.5 is followed by germ cell loss. In the normal course of events, multinucleated cells are detected by the tetraploidy checkpoint, resulting in G1 arrest followed by apoptosis (reviewed in (54)), and germ cells that remain within the interstitium are removed by resident macrophages (55). Therefore, we propose that in both instances, germ cells are lost through apoptotic events designed to remove aberrant and mislocated cells from the germ cell pool. What underlies these phenotypes will be of interest for understanding quality control in the male germline and its vulnerability to environmental stress.

Bi- and multi-nucleated cells can arise during conditions of disease and stress from incomplete cytokinesis, cell-cell fusion, and cell cycle disruptions such as mitotic slippage and endoreduplication. Male germ cells uniquely undergo regulated incomplete cytokinesis and remain connected by stable intracellular bridges forming germline syncytia (56). By E15.5, murine male germ cells are typically quiescent (57) and they do not undergo cytokinesis. Endoreduplication, or genome replication in the absence of mitosis, is due to modulation of cyclin-dependent kinase (CDK) activity (58). In the E13.5 *Inha* KO testis, *Cdkn1c* (encoding p57) and *Cdk18* are both significantly decreased, and intriguingly, *Cdkn1c* is a potent negative regulator of cell proliferation (59). Therefore, we speculate that the decrease in cyclin levels results in endoreduplication and the transient formation of multi-nucleated cells in *Inha* KO testes. The failure to form stable intercellular bridges as a cause of multinucleated cell formation is unsupported by our transcriptional data, as *Tex14*, *Cep55*, and *Anln* are not significantly altered. The driver of this phenotype remains to be determined.

The finding of germ cells outside the cords at E15.5 in *Inha* KO testes is highly unusual, given that macrophages engulf both germ and Sertoli cells outside of the cords as early as E11.5 (55). Both F4/80+ (macrophages) and Ly6G+ (neutrophils) cells are detected inside cords adjacent to germ cells at E13.5 through to PND0 (14), so it is plausible that their number or function within the developing testis is affected by activin A, a known modulator of immune cells in adult testes (60). In addition, factors that compromise the cord basement membrane or enhance germ cell motility could underlie this phenotype. Although the mechanisms regulating initial testis cord formation appear fully functional, with germ cells enclosed by Sertoli cells at E13.5, changes in cord area and number measured at this age demonstrate that exposure to unopposed activin A disturbs this process to some degree. Following initial cord formation, cord structures are actively maintained by Sertoli, peritubular myoid, Leydig and immune cells (61). Although many genes with known roles in cord morphogenesis were not altered in our model (*Wt1*, *Ctnnb1*, *Sox8*, *Sox9*, *Gpr56*, *Stim1*, *Nr0b1*, *Col4a1*, *Col4a2*), transcripts encoding the type IX collagen, *Col9a3*, were decreased prematurely in E15.5 *Inha* KO testes when germ cells are present in the interstitium. This Sertoli cell-expressed gene (62) is negatively regulated by activin A (19). The functional relevance remains speculative, as to our knowledge, *Col9a3* KO mice exhibit no apparent abnormalities of testicular function, although abnormal seminal vesicle morphology has been indicated (https://www.informatics.jax.org/vocab/mp_ontology/MP:0002059). The function of CCL17 exclusively transcribed in Sertoli cells of the fetal testis (48, 63), is unknown, however its role in priming dendritic cell migration (64) suggests that its upregulation in the *Inha* KO testis may enhance inappropriate germ cell movement.

This study identified phenotypes in fetal mouse testes that are common to male mice exposed *in utero* to unopposed activin A and to various EDCs (DEHP, MEHP, DBP), including altered seminiferous cord formation and the presence of multinucleated gonocytes. Both activin A and MEHP, a phthalate ester, promote reduced germ cell numbers and disrupt steroidogenesis during fetal life (27). Phthalate exposure can also increase the incidence of hypospadias and cryptorchidism (50, 65), though this has not been observed in *Inha* KO mice. Fetal Sertoli cells may be particularly susceptible to both activin A levels and to EDCs; chronic DBP exposure in fetal mice changes cord profiles and decreases Sertoli cell number in adulthood (66); the gap junction transcript, *Gja1*, is lowered in rat testes by DEHP (67) but is increased in isolated PND6 mouse Sertoli cells by activin A (68). Together these data strongly indicate that EDCs and activin A each generate multiple TDS phenotypes, affecting the same cell types and processes central to fetal testicular development through common pathways. Interestingly, the combined treatment with activin and MEHP in this study reduced the proportion of gonocytes outside the cords but yielded a similar proportion of MNGs, when compared to exposure to either factor alone. Transcriptional analyses of a limited number of somatic and germ cell genes identified two Sertoli cell specific transcripts, *Amh* and *Ccl17*, affected by both activin and MEHP. *Amh* was reduced in each treatment group, while *Ccl17* levels are increased by activin A and by MEHP, indicating that several aspects of Sertoli cell number and/or function are impaired by altered activin A and by phthalate exposure. The importance of AMH and activin B in maintenance of masculinised supporting cells affects the Sertoli cell lineage between E12.5 and E15.5 (69). It is thus apparent that the unopposed/elevated activin A levels in the *Inha* KO similarly acts on Sertoli cells in this same window of sensitivity, fitting with the observation of activin receptor transcripts in Sertoli lineage cells, and demonstrated by our analysis of *Inhba* KO mouse testes (19). The potential for endocrine disruption to further exacerbate the outcomes of inappropriate modulation of activin levels during this window of gonad development presents an important avenue for investigation.

Overall, comparison of testes from mice with unopposed activin A and those lacking activin A reveals several opposing phenotypes that highlight the importance of optimal bioavailability of this growth factor. This study has demonstrated that there is a progression of outcomes in the fetal mouse testis which are evident immediately after sex determination through to birth, with both somatic and germ cell populations affected. Ingenuity Pathway Analysis identification of factors that may influence the outcome of altered activin A bioactivity levels provides a starting point for examining potential interactions with therapeutics and physiological conditions. How the substantial loss of germ cells in PND0 *Inha* KO testes affects initiation and establishment of postnatal spermatogenesis is currently under investigation.

## Data availability statement

The data presented in the study are deposited in the GEO repository, accession number GSE236618.

## Ethics statement

The animal study was approved by Monash University Animal Ethics Committee. The study was conducted in accordance with the local legislation and institutional requirements.

## Author contributions

PW and KL conceived and designed the study. PW and ML performed experiments. PW, ML, LO’D, DH, KL analyzed and interpreted data. PW, ML, KL wrote first draft. PW, LO’D, KL reviewed and edited manuscript. KL funded project. All authors read and approved the final version of the manuscript.
